# The non-random patterns of genetic variation induced by asymmetric somatic hybridization in wheat

**DOI:** 10.1186/s12870-018-1474-3

**Published:** 2018-10-17

**Authors:** Mengcheng Wang, Yujie Ji, Shiting Feng, Chun Liu, Zhen Xiao, Xiaoping Wang, Yanxia Wang, Guangmin Xia

**Affiliations:** 1 The Key Laboratory of Plant Cell Engineering and Germplasm Innovation, Ministry of Education, School of Life Science, Shandong University, 27 Shandanan Road, Jinan, Shandong 250100 People’s Republic of China; 2 College of Veterinary Medicine, Nanjing Agricultural University, Nanjing, 210095 China; 3 Shijiazhuang Academy of Agriculture and Forestry Sciences, Shijiazhuang, 050041 China

**Keywords:** Introgression line, Genomic shock, Genetic variation, Nucleotide substitution, Insertion and deletion

## Abstract

**Background:**

Asymmetric somatic hybridization is an efficient crop breeding approach by introducing several exogenous chromatin fragments, which leads to genomic shock and therefore induces genome-wide genetic variation. However, the fundamental question concerning the genetic variation such as whether it occurs randomly and suffers from selection pressure remains unknown.

**Results:**

Here, we explored this issue by comparing expressed sequence tags of a common wheat cultivar and its asymmetric somatic hybrid line. Both nucleotide substitutions and indels (insertions and deletions) had lower frequencies in coding sequences than in un-translated regions. The frequencies of nucleotide substitutions and indels were both comparable between chromosomes with and without introgressed fragments. Nucleotide substitutions distributed unevenly and were preferential to indel-flanking sequences, and the frequency of nucleotide substitutions at 5′-flanking sequences of indels was obviously higher in chromosomes with introgressed fragments than in those without exogenous fragment. Nucleotide substitutions and indels both had various frequencies among seven groups of allelic chromosomes, and the frequencies of nucleotide substitutions were strongly negatively correlative to those of indels. Among three sets of genomes, the frequencies of nucleotide substitutions and indels were both heterogeneous, and the frequencies of nucleotide substitutions exhibited drastically positive correlation to those of indels.

**Conclusions:**

Our work demonstrates that the genetic variation induced by asymmetric somatic hybridization is attributed to both whole genomic shock and local chromosomal shock, which is a predetermined and non-random genetic event being closely associated with selection pressure. Asymmetric somatic hybrids provide a worthwhile model to further investigate the nature of genomic shock induced genetic variation.

**Electronic supplementary material:**

The online version of this article (10.1186/s12870-018-1474-3) contains supplementary material, which is available to authorized users.

## Background

Crop species have a lower genetic base or diversity, given anthropogenic selection applied during domestication and improvement processes. Their wild relatives retain genetic diversity, and therefore, are a valuable genetic resource for crop breeding via introgressing genetic materials into crops. Besides remote sexual hybridization [1, 2], genetic manipulation can be applied via somatic hybridization (where somatic protoplasts are induced to fuse, followed by in vitro regeneration). This is especially true when viable remote sexual hybrids are difficult, or impossible, to establish [3]. Asymmetric somatic hybridization is a refined approach, in which donor protoplasts are irradiated to fragment the genome prior to fusion. Thus, most donor chromatin is eliminated, very small amounts of chromatin fragments are introgressed into the recipient genome [4, 5]. The introgression of donor chromatin segments occur via end-joining of fragments, most easily during mitosis [6]. This event leads to a strong genomic shock, the force of genomic variation during natural evolution and diploydization of polyploydies [7, 8], and therefore induces genome-wide genetic variation, which accounts for the agricultural traits of somatic hybrids [6]. However, chromosome rearrangement and large fragment deletion, the characteristic events during diploidization of allopolyploidies, seldom happen in asymmetric somatic hybrid cells given that the contribution of the donor’s genome is largely reduced [3].

We previously generated many wheat asymmetric somatic hybrids with bread wheat cultivar JN177 (modest salt tolerance) as the recipient and tall wheatgrass (*Thinopyrum elongatum*, wheat’s close relative with topmost salt tolerance) as the donor, with aim to introgressing salt-tolerance associated genetic materials into wheat genome. A few derivatives being introgressed with five ~ seven chromosomal fragments of tall wheatgrass were selected based on favorable phenotypes [3, 5, 9–11], some of which are qualified to be released as novel cultivars [9–11]. One of these is the line II-1-3, which was bred to a cultivar SR3 with improved salt and drought tolerance [12], whose genome possesses six observed exogenous fragments [10, 13]. The genomes of these derivatives were found to take place high frequency of genetic variation via molecular marker assays and sequence comparison [10, 14–17]. Note that the genetic variation was largely induced by genomic shock during asymmetric somatic hybridization, because the effect of other factors, such as protoplast isolation, UV radiation, callus induction and plant regeneration was certainly slight [10, 14]. However, these findings have not addressed the fundamental questions concerning such genetic variation. Firstly, do the introgressed fragments induce stronger genetic variation in local chromosomes? Secondly, exogenous fragments are randomly inserted into the recipient chromosomes, then whether the genetic variation is a random or predetermined genetic event? To explore these questions will deepen our insight into the characteristics and difference of genetic variation in somatic hybrids and polyploidies as wee as the genetic basis of their phenotypic alteration from parents.

We have proved that the genetic variation had similar frequency and pattern among SR3 and other introgression lines, and for each introgression line, the genetic variation is genetically stable among different generations of progenies, indicating that asymmetric somatic hybridization-induced genetic variation exhibits the same behavior and mechanism [10]. Our previous study precisely revealed that six chromatin fragments of tall wheatgrass are introgressed into six chromosomes of SR3 genome using the GISH assay (but the sizes and sequences of these introgressed fragmens is still unknown) [5], so SR3 is suitable to address the fundamental questions mentioned above. Here, we used the unigenes of SR3 and its parent wheat JN177 that were previously sequenced via large-scale EST sequencing [17], and found that asymmetric somatic hybridization induced genetic variation through both whole genomic shock and local chromosomal shock, which is a predetermined non-random genetic event.

## Results

### Coding sequences had lower genetic variation rate than non-coding regions

We previously found that asymmetric somatic hybridization induced high frequency of genetic variation in wheat in a genetically stable manner [12, 14–16]. Given that the extent and pattern of genetic variation was similar among introgression lines with different traits [10], here we selected SR3 to uncover the characteristics of this genetic variation through comparing SR3 and JN177 unigene sequences that we previously sequenced [17]. Note that the aim was to know the effect of asymmetric somatic hybridization on wheat genome, so we did not analyze the sequences of the donor parent wheatgrass. Briefly, we got 9634 and 7107 unigenes from SR3 and JN177, respectively. These unigenes were randomly mapped to 21 chromosomes by blasting against wheat survey database, showing that they can mirror the whole genome although the unigenes could not cover all genes, and the data can outline the characteristics of genetic variation induced by asymmetric somatic hybridization.

**Fig. 1:**
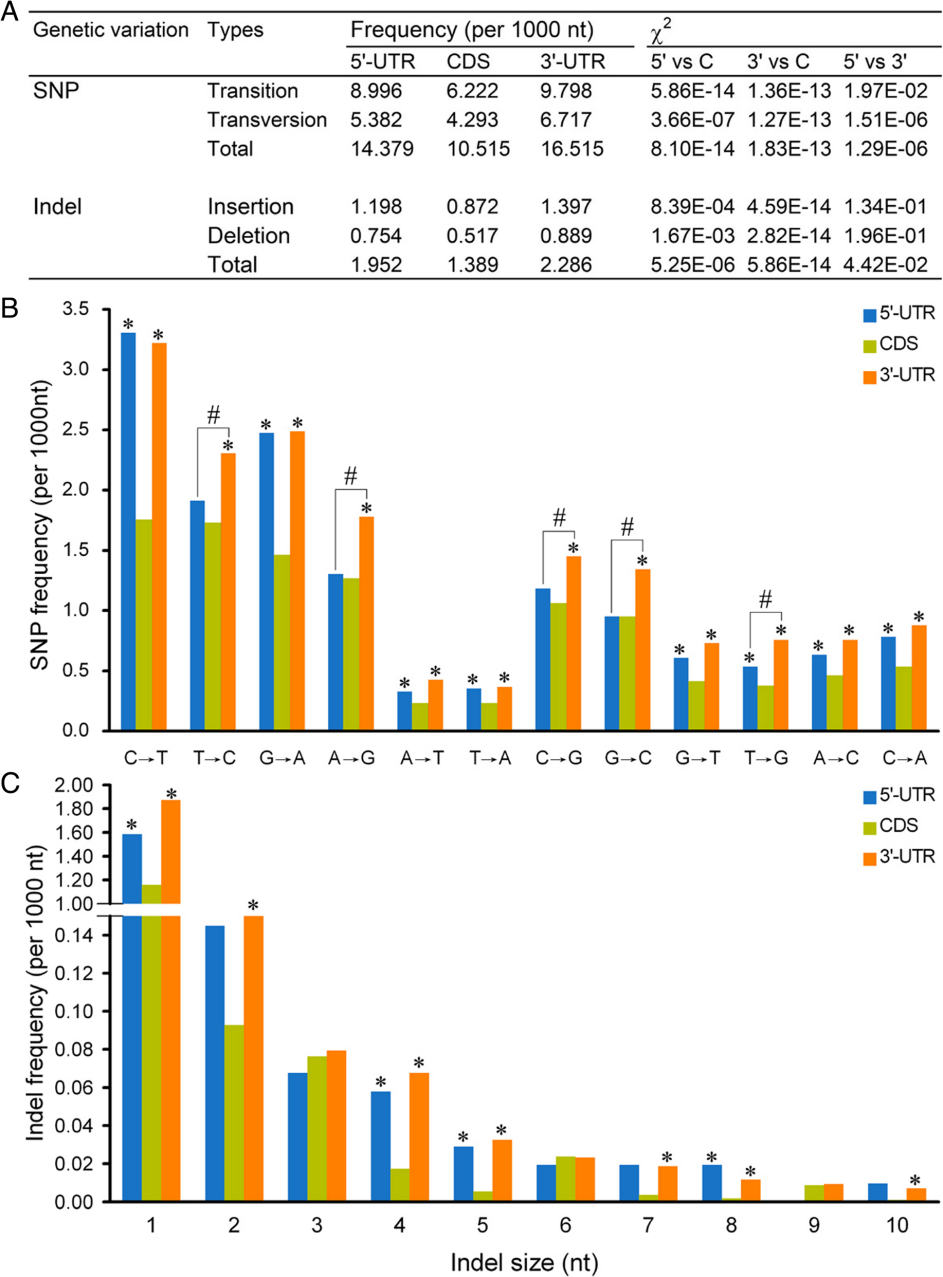
Coding region has lower SNP and indel frequencies than un-translated region. **a**: The total SNP and indel frequencies in 5’-UTR, CDS and 3’-UTR. The difference of the frequencies among three regions was compared using the chi-square test of fourfold cross-table analysis. 5′: 5’-UTR, 3′: 3’-UTR, C: CDS. **b**: The frequencies of twelve types of SNPs in 5’-UTR, CDS and 3’-UTR. **c**: The frequencies of indels with sizes form 1 to 10 nt in 5’-UTR, CDS and 3’-UTR. In (**b**) and (**c**), significant difference between CDS and 5′/3’-UTR (*) as well as between 5′ and 3’-UTR (#) was measured using the chi-square test of fourfold cross-table analysis

Firstly, we analyzed the distribution of SNPs and indels in coding and non-coding regions (Fig. 1a). SNP frequency (10.515) of CDS was significantly lower in comparison with 5′- and 3’-UTRs (*P* = 8.10E-14 and 1.83E-13). The frequency of 3’-UTR (16.515) was substantially higher compared with 5’-UTR (14.379) (*P* = 1.29E-06). Most of twelve types of substitutions had higher frequencies in 5′- and 3’-UTR in comparison with CDS (Fig. 1b). Notably, C → T and G → A frequencies of both 5′- and 3’-UTR were higher by approximately one fold in comparison with CDS (*P* < 8.16E-14).

Insertions were more pronounced than deletions in CDS, 5′- and 3’-UTR (*P* = 0.001 ~ 4.09E-45) (Fig. 1a). In comparison with CDS, 5′- and 3’-UTR had remarkably higher indel frequencies; indel frequency of 3’-UTR were the highest (*P* = 0.002 ~ 5.25E-6 in CDS vs 5’-UTR, and 2.82E-14 ~ 5.86E-14 in CDS vs 3’-UTR). For non-3n indels (non-multiple of three nt), the frequencies of 5′- and 3’-UTR were both higher than that of CDS (*P* = 0.028 ~ 4.76E-14; the exceptions were 7 nt and 10 nt). However, for 3n indels (multiple of three nt), the frequencies were comparable among CDS, 5′- and 3’-UTR (*P* > 0.783). As for indels with sizes greater than 2 nt, 3n indels (3, 6, 9 nt) had higher frequencies than the adjacent non-3n indels (4/5, 7/8, 10 nt, respectively) in CDS (*P* = 0.002 ~ 2.07E-19). Similar results were found when comparing the frequencies of insertions and deletions (*P* = 0.021 ~ 1.80E-14) (Additional file 1: Figure S1). However, the pattern was not present in 5′ and 3’-UTR (*P* > 0.414) (Fig. 1c; Additional file 1: Figure S1).

### Chromosomes with and without exogenous fragments had similar genetic variation

**Fig. 2:**
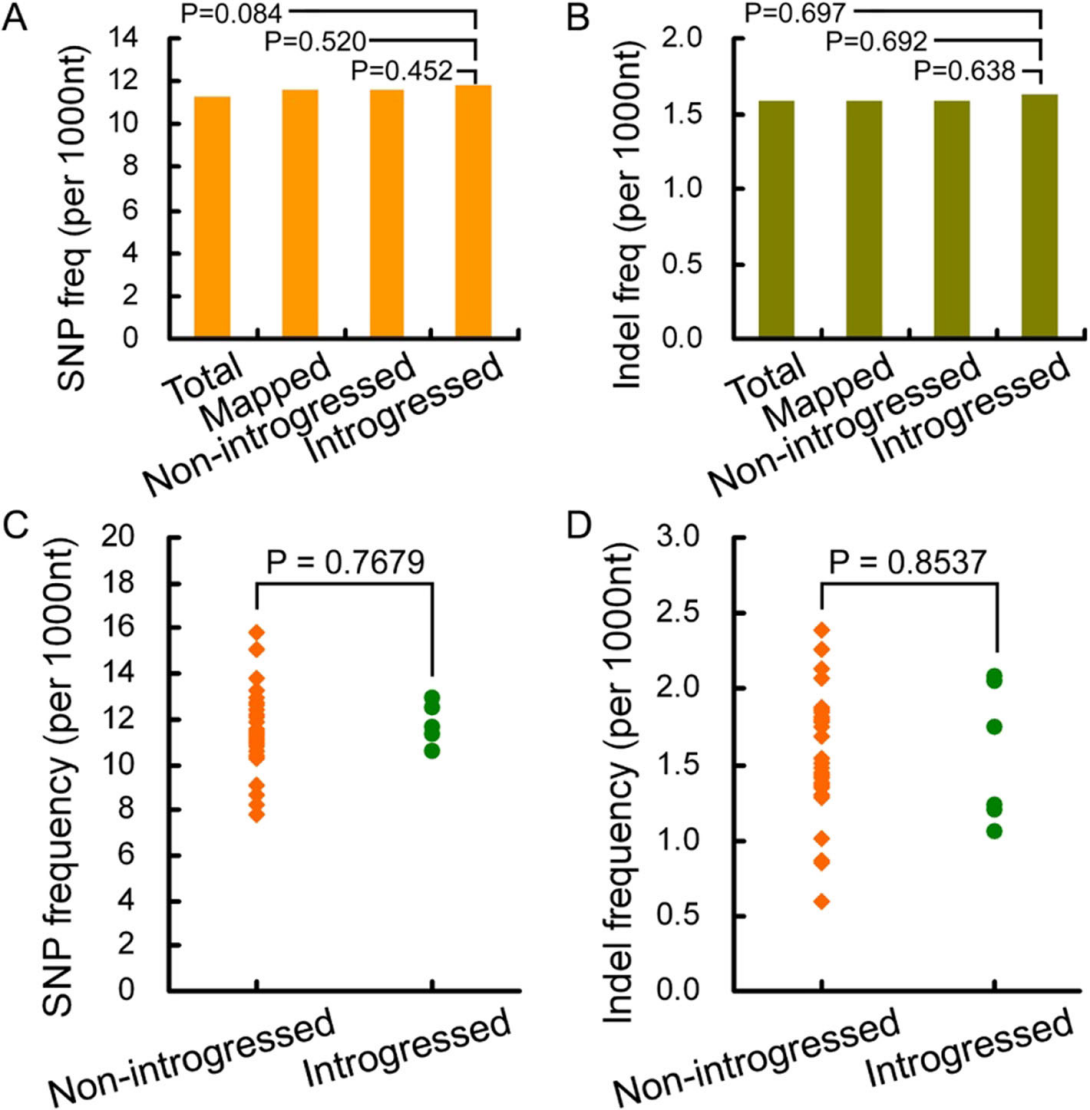
The introgression of exogenous fragment does not induce stronger genetic variation of local chromosomes. **a**: The comparison among SNP frequencies. **b**: The comparison among indel frequencies. **c**: SNP frequencies of chromosomal arms with and without introgressed exogenous fragments. **d**: Indel frequencies of chromosomal arms with and without introgressed exogenous fragments. Total: all unigenes; Mapped: unigenes mapped to different chromosomal arms; Non-introgresed: unigenes mapped to chromosomal arms without exogenous fragments; Introgressed: unigenes mapped to chromosomal arms introgressed with exogenous fragments. In (**a**) and (**b**), *P* values were calculated using the χ^2^ test. In (**c**) and (**d**), *P* values were obtained via the Student’s *t*-test

SR3 genome has six exogenous fragments introgressing in chromosomes 1BL, 1DL, 2AL, 2DL, 5BS, and 6DS, respectively [5]. To know whether exogenous fragments induced stronger genetic variation in introgressed chromosomes, we mapped the unigenes to different chromosomal arms. SNP frequency of unigenes mapped to chromosomal arms introgressed with exogenous fragments (namely introgressed unigenes) was comparable to those of all unigenes (namely total unigenes), unigenes mapped to all chromosomes (namely mapped unigenes), and unigenes mapped to chromosomal arms without exogenous fragments (namely non-introgressed unigenes) (*P* > 0.084) (Fig. 2a). Introgressed unigenes also had similar indel frequency to the other three classes of unigenes (*P* > 0.638) (Fig. 2b). The frequencies of SNP and indels were various among chromosomal arms (coefficient of variation (CV) = 0.15 and 0.25, respectively) (Fig. 2c, d). SNP frequencies were comparable between non-introgressed and introgressed unigenes (*P* = 0.768) (Fig. 2c). Indel frequencies of introgressed unigenes were also in the range of non-introgressed unigenes (*P* = 0.854) (Fig. 2d). As were also found based on the frequencies of transitions, transversions, insertions and deletions (*P* > 0.606) (Additional file 1: Figure S2). These data indicate that the genetic variation occurred unevenly among chromosomes, and the introgression of exogenous fragments did not induce stronger genetic variation in local chromosomes.

### Nucleotide substitutions were positively correlative to indels in chromosomes with introgressed fragments

**Fig. 3:**
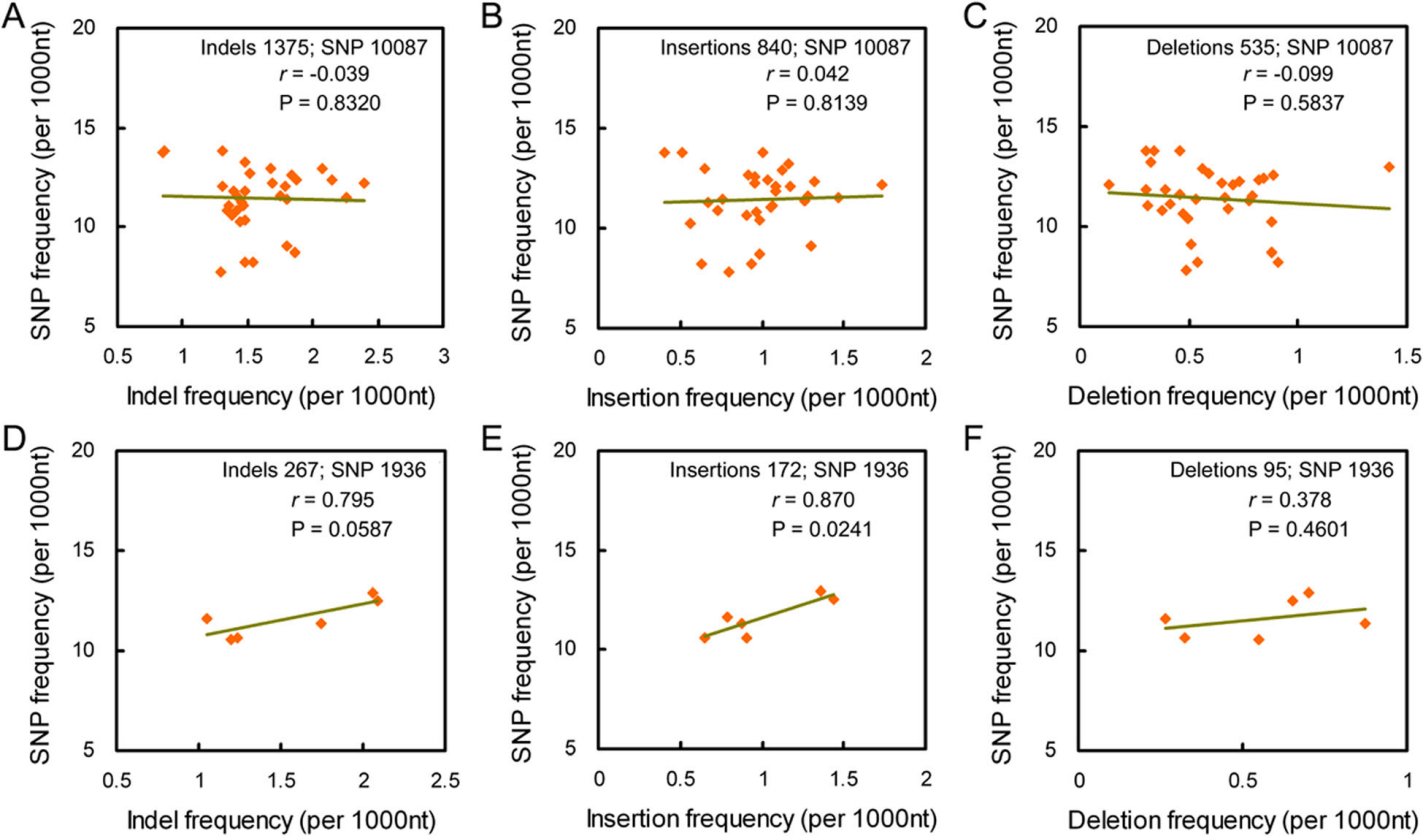
Nucleotide substitutions are correlative to indels in chromosomes introgressed with exogenous fragments. **a**-**c**: The correlation of SNP to indel, insertion and deletion frequencies in chromosomal arms without exogenous fragments. **d**-**f**: The correlation of SNP to indel, insertion and deletion frequencies in chromosomal arms introgressed with exogenous fragments. The correlation was calculated with the Pearson correlation analysis

To know the association of nucleotide substitutions with indels, we analyzed the correlation between their frequencies. There had no correlation based on mapped unigenes (*r* = − 0.175, *P* = 0.274) (Additional file 1: Figure S3a) and non-introgressed unigenes (*r* = 0.039, *P* = 0.832) (Fig. 3a). As was also found between SNP frequencies and insertion or deletion frequencies (|*r*| < 0.120, *P* > 0.466) (Additional file 1: Figure S3b, c; Fig. 3b, c). In introgressed unigenes, there had a positive correlation between SNP and indel frequencies (*r* = 0.795, *P* = 0.059) (Fig. 3d). The correlation was more obvious when SNP and insertion frequencies were compared (*r* = 0.870, *P* = 0.024) (Fig. 3e), but became weaker between SNP and deletion frequencies (*r* = 0.378, *P* = 0.460) (Fig. 3f). These results indicate that nucleotide substitution and indel occurred independently in non-introgressed chromosomes, but had a positive co-effect in introgressed chromosomes.

### Chromosomes with introgressed fragments had more nucleotide substitutions in indel-flanking sequences

**Fig. 4:**
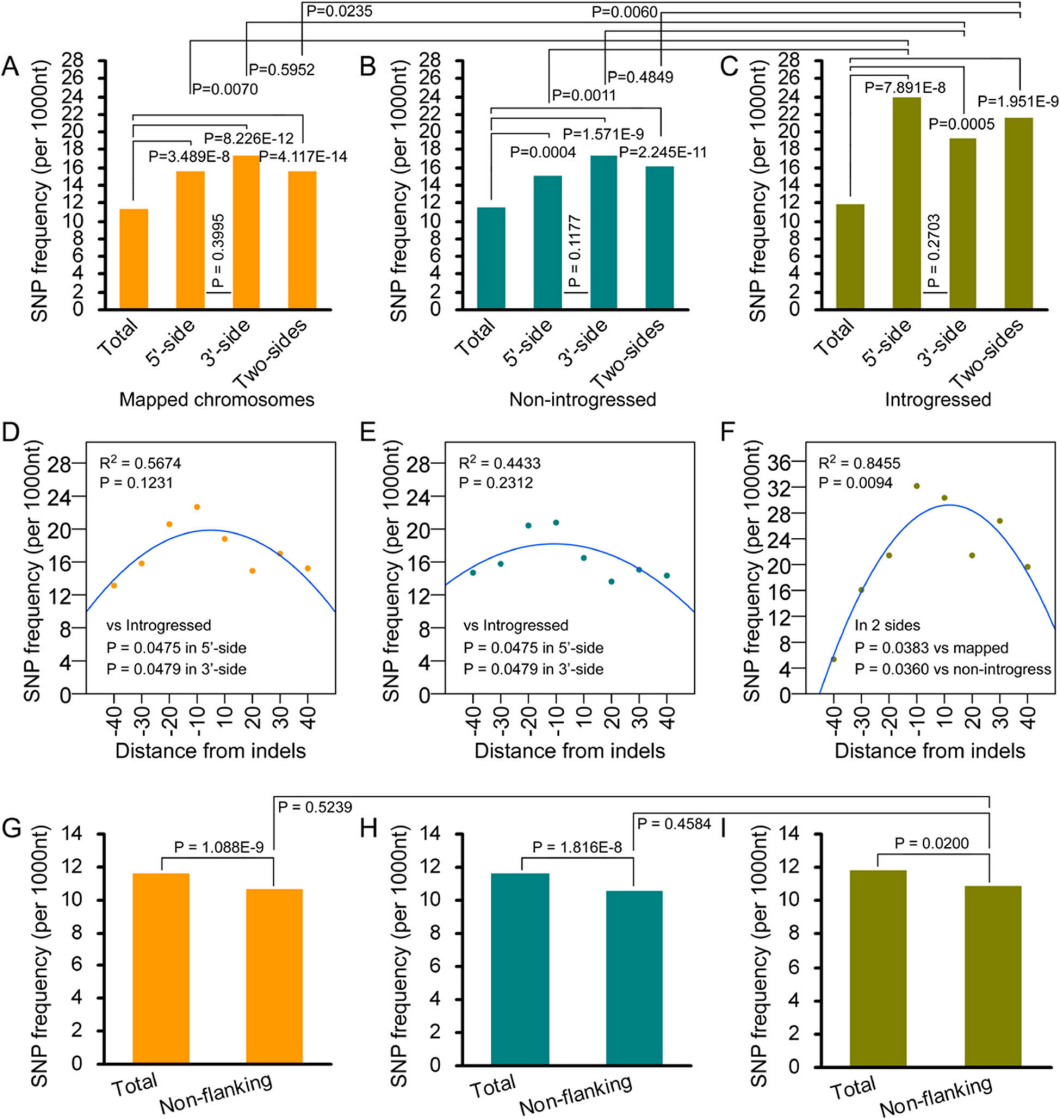
Nucleotide substitution has higher rate close to indels. **a**-**c**: SNP frequencies of indel-flanking sequences. **d**-**f**: SNP frequencies of flanking sequences with different distance from indels. 10, 20, 30, 40: 1–10, 11–20, 21–30, 31–40 nt 3′-flanking sequences of indels; − 10, − 20, − 30, − 40: 1–10, 11–20, 21–30, 31–40 nt 5′-flanking sequences of indels. **g**-**i**: SNP frequencies of non-flanking sequences of indels. In (**a**)-(**c**) and (**g**)-(**i**), the difference significance was analyzed by the χ^2^ test. In (**d**)-(**f**), the difference in change of SNP frequencies among intervals of flanking sequences was compared with the paired *t*-test. **a**, **d** and **g**: unigenes mapped to all chromosomal arms; **b**, **e** and **h**: unigenes mapped to chromosomal arms without exogenous fragments; **c**, **f** and **i**: unigenes mapped to chromosomal arms introgressed with exogenous fragments. In **e**-**f**: The curve was charted based on the quadratic regression equation through the regression analysis

To clarify the cause for positive correlation between nucleotide substitutions and indels in introgressed chromosomes, SNP frequencies of flanking and remote sequences of indels were calculated. In mapped and non-introgressed unigenes, two-sides of flanking sequences had higher SNP frequency than the whole sequences (*P* < 0.0004), and 3′-flanking sequences had slightly higher frequency than 5′-flanking sequences (*P* = 0.400 and 0.118) (Fig. 4a, b). In introgressed unigenes, SNP frequency of two-side flanking sequences were nearly two fold to that of whole sequences (*P* < 0.0005) (Fig. 4c). However, opposite to non-introgressed unigenes, 5′-flanking sequences had slightly higher SNP frequency than 3′-flanking sequences in introgressed unigenes (*P* = 0.270). When compared to either mapped or non-introgressed unigenes, SNP frequencies of both 5′- and two-side flanking sequences were significantly higher (*P* = 0.001~ 0.024), while the frequency of 3′-flanking sequences was similar (*P* = 0.595 and 0.485) (Fig. 4a-c).

The effect of distance from indels was further detected by separating flanking sequences into 10 nt intervals. SNP frequencies increased following the decrease in distance from indels, and the trend appeared to be more obvious in 5′- than in 3′-flanking sequences (Fig. 4d-f). The difference in change of SNP frequencies among intervals in two sides of flanking sequences was more distinguishable in introgressed unigenes than in mapped and non-introgressed unigenes (*P* = 0.038 and 0.036). Similar result was found when both 5′- and 3′-flanking sequences was calculated (*P* = 0.048). In 5′-flanking sequences, SNP frequency was 32.14 in 1-10 nt 5′-flanking sequences, but decreased to 5.36 in 31-40 nt sequences (Fig. 4f); the two frequencies were 22.69 and 13.13 in mapped unigenes, and 20.79 and 14.70 in non-introgressed unigenes (Fig. 4d, e). As a result, SNP frequencies were correlative to distance from indels, but the correlation was more significant in introgressed unigenes (*R*^2^ = 0.846, *P* = 0.009) than in mapped and non-introgressed unigenes (*R*^2^ = 0.567 and 0.443, *P* = 0.1231 and 0.2312) (Fig. 4d-f).

Opposite to flanking sequences, SNP frequencies of sequences remote from indels (namely non-flanking sequences) were significantly lower compared to the whole sequences (*P* = 0.020~ 1.09E-9) (Fig. 4g-i). In comparison to mapped and non-introgressed unigenes, SNP frequency of non-flanking sequences in introgressed unigenes had no significant difference (*P* = 0.524 and 0.458) (Fig. 4g-i). These results indicate that nucleotide substitution distributed unevenly, and preferred to sequences adjacent to indels, especially in chromosomes with exogenous fragments.

### Seven groups of allelic chromosomes had comparable genetic variation

**Fig. 5:**
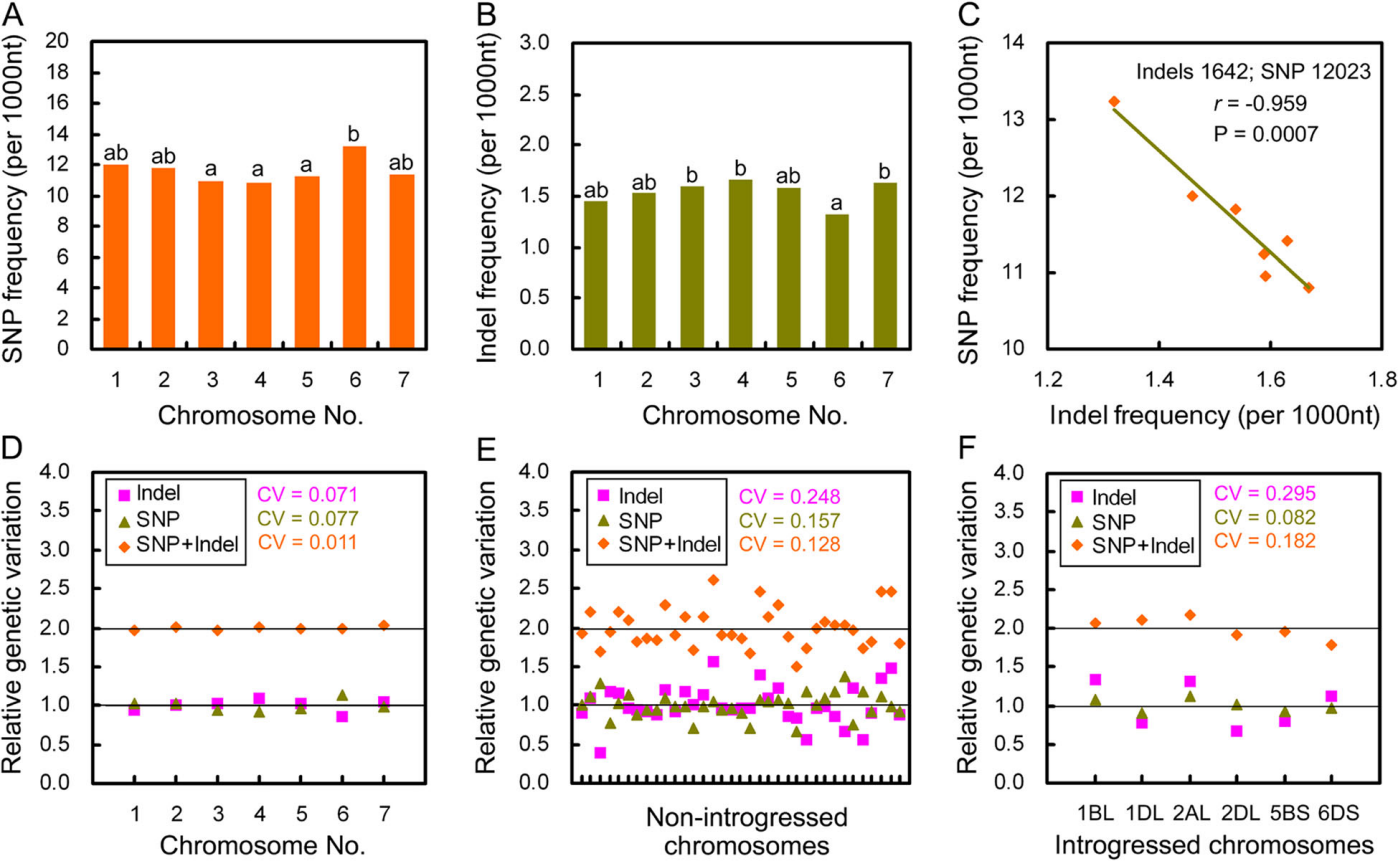
Seven groups of allelic chromosomes had similar genetic variation and exhibited negative correlation between nucleotide substitutions and indel frequencies. **a**, **b**: SNP and indel frequencies of all unigenes mapped to seven allelic chromosomes. **c**: The correlation between SNP and indel frequencies shown in panels (**a**) and (**b**). **d**-**f**: The relative SNP and indel frequencies of unigenes mapped to seven allelic chromosomes (**d**), chromosomes without introgressed fragment (**e**), and chromosomes with introgressed fragments (**f**). In **a** and **b**: The significance of difference was calculated with the the χ^2^ test, and columns labelled with no same letter means the difference is significant (*P* < 0.05). In **c**, the correlation was calculated using the Pearson correlation analysis. In **d**-**f**, CV was coefficient of variation which was calculated as the ratio of standard deviation to mean

Allohexaploid wheat has seven groups of allelic chromosomes originating from A, B and D genomes. In each allelic chromosome group, SNP and indel frequencies of unigenes mapped to long and short arms of allelic chromosomes were various, and their ratios were also diverse (Additional file 1: Figure S4a-c), showing both nucleotide substitutions and indels distributed randomly among allelic chromosomes. When all unigenes in each group were considered together, the groups with higher SNP frequencies had lower indel frequencies (Fig. 5a, b), resulting in a strongly negative correlation between SNP and indel frequencies (*r* = − 0.959, *P* = 6.56E-04) (Fig. 5c). This suggests the extents of total genetic variation are similar among seven allelic chromosome groups. To confirm the suggestion, SNP and indel frequencies were normalized by dividing average SNP and indel frequencies respectively of seven groups, getting relative SNP and indel frequencies. Both relative SNP and indel frequencies fluctuated around 1 with similar fluctuation range (CV = 0.071 and 0.077) (Fig. 5d). In each group, two relative frequencies positioned at two sides of 1 with similar residuals. The sums of relative SNP and indel frequencies were all almost equal to 2 (CV = 0.011), showing total genetic variation was similar among seven allelic chromosome groups. This rule was absent in non-introgressed and introgressed chromosomal arms (Fig. 5e, f). Especially, in introgressed chromosal arms, both relative SNP and indel frequencies were greater or less than 1, coinciding with the positive correlation between SNP and indel frequencies (Fig. 3d). These results indicate that seven groups of allelic chromosomes occurred similar strength of genetic variation, while within each group, genetic variation distributed differently among allelic chromosomes.

### Three genome sets possessed different genetic variation

**Fig. 6:**
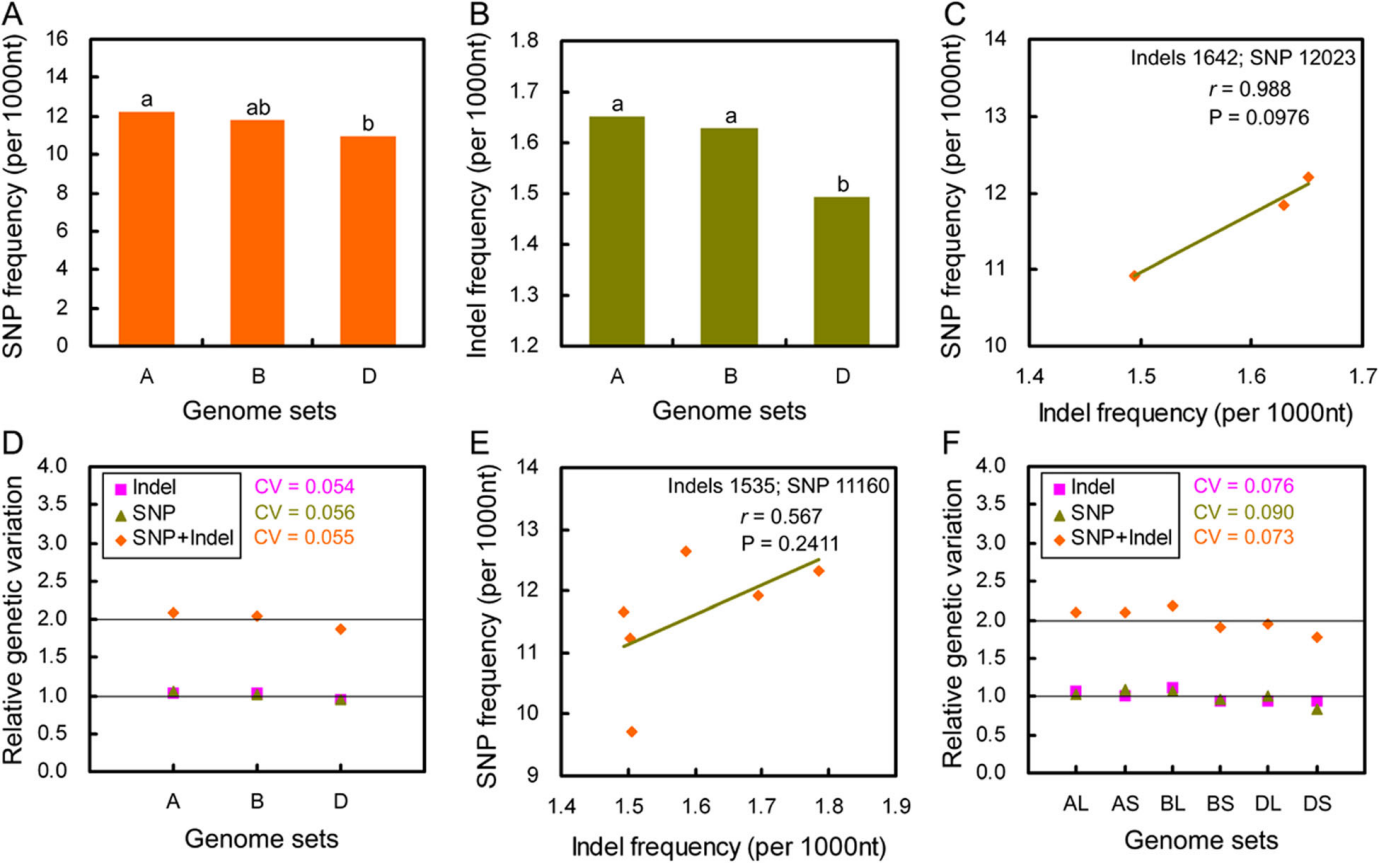
Three genome sets had different genetic variation and exhibited positive correlation between nucleotide substitutions and indel frequencies. **a**, **b**: SNP and indel frequencies of all unigenes mapped to three genome sets. **c**: The correlation between SNP and indel frequencies shown in panels (**a**) and (**b**). **d**: The relative SNP and indel frequencies of unigenes mapped to three genome sets. **e**: The correlation between SNP and indel frequencies of unigenes mapped long and short arms of three genome sets. **f**: The relative SNP and indel frequencies of unigenes mapped to long and short arms of three genome sets. In **a** and **b**: The significance of difference was calculated with the the χ^2^ test, and columns labelled with no same letter means the difference is significant (*P* < 0.05). In **c** and **e**, the correlation was calculated using the Pearson correlation analysis. In **d** and **e**, CV was coefficient of variation which was calculated as the ratio of standard deviation to mean

Alike seven allelic chromosome groups, both SNP and indel frequencies were various among chromosomes in each of three genome sets (Additional file 1: Figure S4d-f). Unigenes from A genome had the highest SNP and indel frequencies, while those from D genome had the lowest frequencies (Fig. 6a, b), so that SNP frequencies were strongly positively correlative to indel frequencies (*r* = 0.988, *P* = 0.098) (Fig. 6c). The relative SNP frequency was almost equal to the relative indel frequency in each genome set, and the relative SNP and indel frequencies as well as their sums exhibited comparable difference among three genome sets (CV = 0.054~ 0.056) (Fig. 6d). When calculated on basis of chromosomal arms, the positive correlation between SNP and indel frequencies was weakened (*r* = 0.567; *P* = 0.241) (Fig. 6e); while the relative SNP frequencies were still similar with the relative indel frequencies, and their sums were obviously distinct from each (Fig. 6f). These results indicate that sequences from different ancestors occurred different extent of genetic variation.

## Discussion

### Asymmetric somatic hybridization-induced genetic variation is associated with selection pressure

Genetic variation serves as an evolution driver, and is affected by selection pressure during plant evolution. Thus, CDS is under stronger selection pressure compared with UTR [18], and indels with sizes of multiples of three not resulting in frameshift mutation suffer less selection pressure [19], because indels are expected to be deleterious when they occur in functional sequences, especially coding regions where frameshift can be induced [20]. Here, the genetic variation frequencies were lower in CDS than in UTR, and 3n-indels had higher frequencies compared to non-3n indels with adjacent sizes in CDS but not in UTR (Fig. 1; Additional file 1: Figure S1). In line with these data, we suggest genetic variation being stably reserved in SR3 is under selection pressure. Given that the introgression lines with different agricultural traits has similar frequency of genetic variation [10], and salt-tolerant genes have comparable frequencies of genetic variation to other genes between SR3 and JN177 [17], we believe that the selection seems not to be associated with agricultural traits, and the change of agricultural traits is the consequence genetic variation in the genomes of introgression lines.

The alteration of cytosine methylation was found in the genomes of allopolyploidies [21–24], newly synthesized allohexaploid wheat [21] and wheat asymmetric somatic hybrids [10, 25], so epigenetic modification is a common consequence of genomic shock. Epigenetic regulation of gene transcription is one aspect of genomic asymmetry during diploidization of allopolyploidies [26]. We previously found that asymmetric somatic hybridization significantly alters cytosine methylation profiles [25]. As methylated cytosines are readily converted to thymine [27], DNA methylation represents a major source of SNP formation (C → T, and G → A in complementary strand) [28]. Here, the frequencies of C → T and G → A in 5’-UTR were significantly higher than those in CDS in SR3 vs JN177 comparison (Fig. 1b), showing that epigenetic modification mediated nucleotide substitution is one of the major forces of genetic variation induced by asymmetric somatic hybridization. Moreover, the difference in DNA methylation profiles partially accounts for the differential expression of salt-associated genes between SR3 and JN177 [25]. Together, epigenetic variation may play crucial roles in asymmetric somatic hybridization-induced introgression lines.

### Asymmetric somatic hybridization leads to whole genomic shock and local chromosomal shock

Genomic shock has been acted as the inducer of genetic variation during various events such as natural evolution and dipolyploidization of polyploidies [7, 8]. Our previous study also found that the high frequency of genetic variation in wheat introgression lines was attributed to genomic shock [10]. In asymmetric somatic hybrids, introgressed segments deserve to lead to stronger genomic shock on local chromosomes than the other chromosomes. However, both nucleotide substitution and indel frequencies had no difference between chromosomes with and without exogenous fragments (Fig. 2), indicating that the introgression of exogenous fragments predominantly leads to whole genomic shock so that high frequency of genetic variation is induced at whole genome scale. A possible cause is that donor chromatin segments are introgressed via the mechanism of end-joining of fragments, which is mutagenic and therefore a less preferred mechanism as it usually results in point mutations and deletions of various size during repair [29]. On the other hand, except for six visible introgressed fragments, small introgressed fragments that could not be detected with the GISH assay may be present in the genome, because we found that some members of glutenin gene family as well as several genes responsive to salt stress in the wheat introgression lines came from the donor wheatgrass or were the mosaic forms between the homologs of wheat and wheatgrass ([14, 15]; data not shown). These small introgressed fragments also act as the stimulator of genetic variation, because the indels from intermediate-length short to 60 bp to large-length up to 10 Mb can give rise to detectable genomic shock [30–32].

Indel-induced nucleotide substitution preferentially occurs in flanking sequences [20], and substitution level increases close to indels [33, 34]. Here, higher SNP frequency in indel-flanking sequences as well as the increase of SNP frequency close to indels was also found (Fig. 4), providing a direct evidence for that the rule - indel is a local “mutator” [20, 35–37] – is also present in the wheat introgression lines. This rule of “indel-associated polymorphism” gives rise to hot spots of genetic variation, where the frequencies of both indels and SNP are higher than other regions in the genome [38]. However, there has no correlation between SNP and indel frequencies (Fig. 3a-c; Additional file 1: Figure S4), indicating that the rule of “indel-associated polymorphism” does not play major induction effect on nucleotide substitutions at whole genome level in regard of asymmetric somatic hybridization induced genetic variation, which is inconsistent with genetic variation of polyploidies as well as natural vaiation of plants [20, 33, 34].

Note that in chromosomes introgressed with exogenous fragments, SNP frequencies were positively correlative to indel frequencies (Fig. 3d-f). It has proved that indels, especially large indels, locally suppress crossovers [39, 40], and produce topological constraints for homologous pairing increase mutation directly [20, 35–37], which reduce frequency of recombination, and accumulate genetic variation of indel-surrounding sequences. Large-length indels performs the similar effect on genetic variation as to genomic rearrangement that seriously suppress recombination [7]. This implies that visible introgressed fragments may give rise to a local chromosomal shock to induce the occurrence of genetic variation via suppressing the recombination in the local chromosomes. Given the indifference between the frequencies of genetic variation in introgressed and non-introgressed chromosomes (Fig. 2), it could be concluded that local chromosomal shock plays the minor effect on genetic variation, but whole genomic shock has the predominant effect. The interesting issue is whether the frequency becomes higher in the introgressed fragment flanking regions, which could not be measured now because it is difficult to determine the positions of introgressed fragments in the chromosomes. Moreover, 5′-flanking sequences of indels had drastically higher SNP frequencies in the introgressed chromosomes than the other (Fig. 4c, f), which is inconsistent with the finding that the nucleotide substitution increases close to indels in both sides of flanking sequences [20, 33, 34]. The inconsistence reflects the specificity of the mechanism governing indel-associated nucleotide substitution in the genetic variation induced by asymmetric somatic hybridization, which is worthy of being studied in the future.

### Asymmetric somatic hybridization induces genetic variation in a non-random manner

SNPs and small indels are two major natural genetic variation in organisms [41], so their rates determine the extent of genetic variation, and therefore, the strength of selection pressure. Non-allelic chromosomes are generally bound to suffer from similar selection pressure, and therefore bear comparable extent of genetic variation. Here, equal relative genetic variation among seven groups of allelic chromosomes (Fig. 5), indicating non-allelic chromosomes have equilibrious predetermined extent of genetic variation in asymmetric somatic hybrids, and to avoid exceeding this extent, nucleotide substitutions and indels occur in a contradictive manner (Fig. 5) to maintain an intrinsic homeostasis. This phenomenon has not been found up to now in the genetic variation of allopolyploidies and other plants. During dipolyploidization of allopolyploidies, genomic asymmetry caused by genetic variation within allelic loci is strictly controlled [26], highlighting the difference in genetic variation within allelic chromosomes induced by asymmetric somatic hybridization and allopolyploidization. We speculate that the equilibrium of genetic variation is a predetermined event, because the genetic variation in the wheat introgressine lines maintains stability from the generation of somatic hybrids [10].

On the other hand, unequal relative genetic variation was found among three genome sets as revealed by the positive correlation between nucleotide substitution and indel frequencies (Fig. 6). Therefore, unlike non-allelic chromosomes, genetic variation is disequilibrious among genomes coming from three ancestors, which owes to the co-occurrence of both nucleotide substitution and indels. Consistently, genetic diploidization of allopolyploidies is also a non-random but regulatory process [26]. However, opposite to the finding that B genome exhibits a higher marker polymorphism than A genome [26], A genome had the highest genetic variation frequency (Fig. 6), indicating the difference of genetic variation within non-allelic chromosomes induced by asymmetric somatic hybridization and allopolyploidization. Interestingly, the genetic variation frequencies of A and B genomes were higher and their difference was smaller when compared to D genome (Fig. 6). Allohexaploid wheat has evolved through two successive natural hybridizations. The first brought together A and B genome ancestors to form AB allotetraploidy, and the second involved a domesticated form of AB allotetraploidy and D genome ancestor to form bread wheat ABD genome [42]. A wide genomic variation was taken place to achieve diploidization after each natural hybridization, so A and B genomes were suffered from twice genomic variations. This may result in higher endurance threshold of genetic variation in A and B genomes, so that they took place higher frequency of genetic variation than D genome during asymmetric somatic hybridization.

**Fig. 7:**
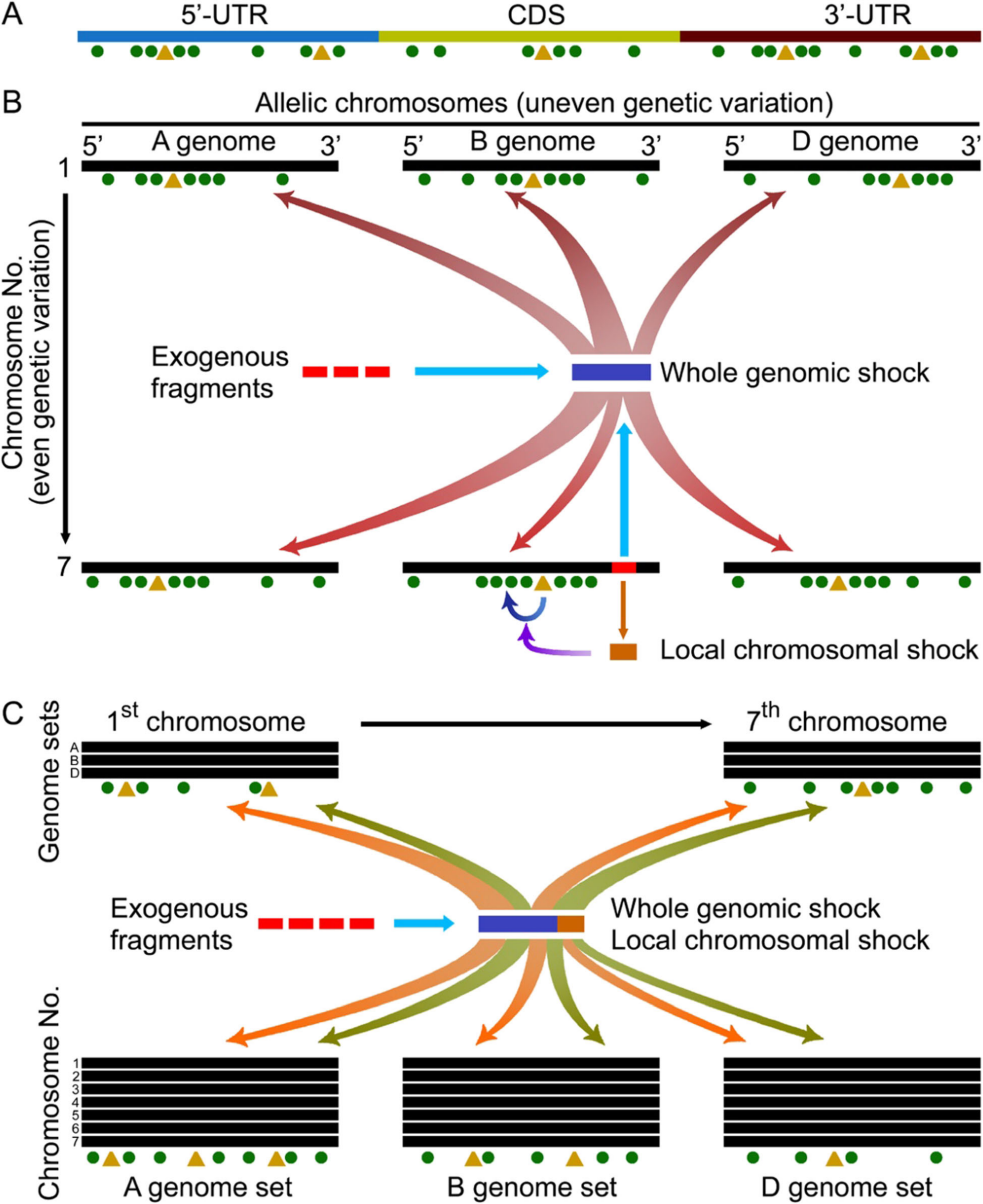
The model of genetic variation induced by asymmetric somatic hybridization. **a**: Untranslated regions (UTR, non-coding sequences) have stronger genetic variation than coding sequences, and the genetic variation distributes unevenly in genes with higher frequency in indel-flanking sequences. **b**: The introgression of exogenous fragments induces genome-wide genetic variation by whole genomic shock and local chromosomal shock. **c**: The introgression of exogenous fragments induces comparable extent of genetic variation among seven allelic chromosomes but different extent among three genome sets. ▲: insertion and deletion. ●: nucleotide substitution. Red block: introgressed exogenous fragments. Blue block: whole genomic shock. Orange block: local chromosomal shock. Red curved arrows: the induction of genetic variation by the whole genomic shock, and the thickness of arrows indicates the strength of genetic variation. Blue curved arrow: the promotion of indels to nucleotide substitution at 5′-flanking sequence. Purple curved arrow: the effect of local chromosomal shock on the promotion of indels to nucleotide substitution at 5′-flanking sequence. Orange and green curved arrows: the induction of indels (orange) and nucleotide substitutions (green) by the whole genomic shock and local chromosomal shock, and the thickness of arrows indicates the strength of genetic variation. The number of dots indicates the frequency of nucleotide substitution, and the distance between dots indicated the distribution of nucleotide substitution

In summary, our work primarily uncovers the behavior of asymmetric somatic hybridization-induced genetic variation. Firstly, the genetic variation distributes unevenly in genes, with lower frequency in coding sequences (Fig. 7a). Secondly, the introgression of exogenous fragments produces both whole genome shock and local chromosomal shock, the former performs the major role to induce high and unequal frequencies of genetic variation in all chromosomes, while the latter has the minor effect to induce nucleotide substitution in sequences, especially 5′-sequences, adjacent to indels in introgressed chromosomes (Fig. 7b). Thirdly, the co-effect of two types of shocks induces equal genetic variation among seven groups of allelic chromosomes by the occurrence of nucleotide substitutions and indels in a negatively correlative manner, but uneven genetic variation among three sets of genomes via the co-occurrence of nucleotide substitution and indel in a positively correlative manner (Fig. 7c). Thus, genetic variation induced by asymmetric somatic hybridization is not a random genetic event. How the genetic variation is determined is a black box worthy of being investigated. Moreover, widespread alteration of DNA sequence, such as point mutants and indels, induced by genomic shock has been observed in de novo wide hybrids and inferred from the analysis of natural allopolyploids [43–45]. The difference and similarity in genetic variation induced by asymmetric somatic hybridization and diploidization of allopolyploidies are also open questions to be answered. Specially, major parts of wheat genome sequences were composed by repetitive elements [46], which are often epigenomic targets that are especially affected by the symmetric hybridization. Thus, following the advance of wheat genome sequencing, we could get deeper insight into the characteristics of asymmetric hybridization-induced genomic variation.

## Conclusions

This work firstly analyzes the genetic behavior of asymmetric somatic hybridization-induced genetic variation. The genetic variation induced by asymmetric somatic hybridization preferentially occurs at hot spots and distributes unevenly within gene sequences. Introgressed fragments lead to both whole genomic shock and local chromosomal shock, inducing genetic variation in a partially different manner between chromosomes with and without introgressed fragments. Genetic variation is equal among non-allelic chromosomes but unequal among genome sets. These data indicate that asymmetric somatic hybridization-induced genetic variation is a predetermined non-random event under selection pressure.

## Methods

### Wheat materials, cDNA library construction, and sequence cleaning

JN177 is a bread wheat cultivar with modest salt and drought tolerance. The salt and drought wheat cultivar SR3 was bred from the introgression lines that were regenerated from the fused cells of the protoplasts of JN177 and wheat’s close relative tall wheatgrass (*Thinopyrum elongatum*) with topmost salt tolerance via the asymmetric somatic hybridization approach [11]. Asymmetric somatic hybridization was achieved through fragmenting the chromatin of tall wheatgrass by UV-irradiation before cell fusion, so most chromatin fragments were eliminated and only several ones were introgressed in the wheat genome. Thus, SR3 is a wheat introgression line with the cultivar JN177 as the recipient and tall wheatgrass as the donor. SR3 took place genome-wide genetic and epigenetic variations [10, 17]. In combination of physiological, transcriptomic and proteomic analysis, the salt and drought tolerance of SR3 is largely attributed to the superior capacities of redox homeostasis maintenance and ionic homeostasis reconstruction [47–49]. Moreover, several important genes involved the processes were identified, including *TaCHP*, *TaOPR1*, *TaAOC1*, *TaSRO1*, and so on, among which most genes have allelic variation in coding sequence or promoter, and *TaSRO1* localizes in the salt tolerant QTL and is the candidate QTL major gene [12, 50–52]. Thus, SR is a special mutant for mining abiotic stress responsive genes. On the other hand, SR3 and other wheat introgression lines took place genome-wide genetic variation in a similar manner, so SR3 can be used to explore the patterns of asymmetric somatic hybridization-induced genetic variation.

JN177 seeds used for generating introgression lines and for EST sequencing were come from the same seed batch to avoid the variation existed before hybridization. The detailed procedure of cDNA library construction and EST sequencing was stated in [17]. Briefly, SR3 and JN177 seedlings under the control, and 200 mM and 18% PEG treatment were selected to extract RNA. RNA samples of each cultivar were pooled to construct cDNA library using a CloneMiner™ cDNA Library Construction Kit (Invitrogen, USA). Two libraries were used for large-scale EST sequencing from 5′-terminal by the Sanger sequencing method.

To gain high quality of sequence is the prerequisite of genetic variation analysis. The detailed method for sequencing cleaning and assembly was presented in [17]. Briefly, the sequences were cleaned on basis of Q20 criteria [53], and highly qualified EST sequences (> 100 nt) were assembled to produce unigenes (overlap 50 nt, identity 95%) [54]. To confirm whether the variation was resulted from sequencing error, we randomly selected a few unigenes with allelic variation to amplify their relevant sequences from cDNAs of SR3 and JN177, and compared the difference of amplicons. The result indicated that these variations were almost all present between SR3 and JN177 (Additional file 1: Figure S5), showing the assembled unigenes were qualified to further analysis.

### Local BLAST

SR3 unigenes were subject to BLASTN [55] against JN177 unigenes (E-value cut-off 1E-10, HSP length cut-off 33). The matched sequences with identity > 96% [to exclude the interference of paralogous genes [56]] were extracted for calculating SNP and indel frequencies. For extracting the 5’-UTR, CDS and 3’-UTR, SR3 unigenes were subject to BLASTX [57] against the non-redundant protein database (ftp://ftp.ncbi.nlm.nih.gov/blast/db/). When a matched peptide started from methionine (Met), the nucleotide sequence before the corresponding start codon ATG was acted as 5’-UTR. As for a matched peptide, in its corresponding nucleotide sequence, when the codon of the last matched amino acid was followed by a stop codon, the sequence after the stop codon was considered as 3’-UTR. The matched sequence after 5’-UTR and/or before 3’-UTR was characterized as CDS. 5’-UTR, CDS and 3’-UTR of SR3 unigenes were subject to BLASTN against JN177 unigenes with above parameters, and the matched sequences with identity > 96% were extracted for analysis.

### Polymorphism calculation

SNP was defined as the conversion of the nucleotide of query sequence (e.g. A) from the nucleotide of subject sequence (e.g. G) as the reference (SNP was G → A). Insertion and deletion were also defined with the subject sequence as the reference: nucleotide fragment present in the query sequence but absent in the subject sequence was considered as an insertion; the opposite was considered as a deletion. SNP and indel frequencies were defined as the ratio of total SNP and indel amounts to the total length of matched regions of all selected sequences with identity > 96%.

### Indel-flanking and indel-remote sequence extraction

50 nt of 5′- and 3′-flanking sequences of indels were extracted for calculating SNP frequency. To avoid the terminal effect, 5′- and 3′-terminal 50 nt sequences were truncated before extracting. To avoid the effect of adjacent indels, sequences between two indels with distance less than 100 nt were excluded. To detect the association between SNP frequency and distance from indels, indel-flanking were separated every 10 nt interval, and the sequences of each interval were extracted. To extract non-flanking sequences of indels, 5′- and 3′-terminal 50 nt sequences were truncated, and then sequences with distance greater than 50 nt to 5′- and/or 3′-indels were extracted.

### Chromosomal localization

The unigenes of SR3 were compared with wheat survey database (http://wheat-urgi.versailles.inra.fr/Seq-Repository) to determine the chromosomal localization. The criteria for chromosomal localization of a unigene were: three topmost matched sequences from wheat survey database had identities > 96%; these three topmost matched sequences were come from the same allelic chromosomes of A, B and D genomes respectively; the sequence with the highest identity of the three matched sequences was selected; the chromosomal localization of the unigene was mapped according to this matched sequence. The unigenes that were mapped to each of chromosomal arms were used for calculating SNP and indel frequencies by BLASTN against JN177 unigenes.

### Statistical analysis

The difference in SNP or indel frequencies among 5’-UTR, 3’-UTR and CDS, as well as between flanking or non-flanking sequence was calculated using the chi-square (χ^2^) test of fourfold cross-table analysis. The difference in total SNP or total indel frequency of unigenes mapped to introgressed chromosomal arms from those of all unigenes, unigenes mapped to all chromosomal arms, unigenes mapped to non-introgressed chromosomal arms were also calculated using the χ^2^ test of fourfold cross-table analysis. The change of SNP frequencies among different intervals of indel-flanking sequences between introgressed unigenes and non-introgressed/mapped unigenes was compared using the paired *t*-test. The difference in SNP or indel frequencies in introgressed chromosomal arms from those in non-introgressed chromosomal arms was measured by the Student’s *t*-test. The association between SNP frequency and distance to indels was analyzed with quadratic regression. The association between SNP and indel/deletion/insertion frequencies was calculated using the Pearson correlation analysis for unigenes mapped to all chromosomal arms and non-introgressed chromosomal arms, and for unigenes mapped to introgressed chromosomal arms, seven allelic chromosomes, and three genomic sets.

## Additional file

Additional file 1: **Figure S1.** The genic distribution of insertions and deletions in unigenes. (A): The frequencies of insertions with sizes form 1 to 10 nt in 5’-UTR, CDS and 3’-UTR. (B): The frequencies of deletions with sizes form 1 to 10 nt in 5’-UTR, CDS and 3’-UTR. Significant difference between CDS and 5′/3’-UTR (labeled *) was measured using the chi-square test of fourfold cross-table analysis. **Figure S2.** The introgression of exogenous fragment does not induce stronger genetic variation of local chromosome. (A): The transition frequencies of chromosomal arms with and without introgressed exogenous fragments. (B): The transversion frequencies of chromosomal arms with and without introgressed exogenous fragments. (C): The insertion frequencies of chromosomal arms with and without introgressed exogenous fragments. (D): The deletion frequencies of chromosomal arms with and without introgressed exogenous fragments. Total: all unigenes; Mapped: unigenes mapped to different chromosomal arms; Non-introgresed: unigenes mapped to chromosomal arms without exogenous fragments; Introgressed: unigenes mapped to chromosomal arms introgressed with exogenous fragments. *P* values were obtained via the Student’s t-test. **Figure S3.** Nucleotide substitutions are not correlative to indels in unigenes mapped to all chromosomes. The correlation was calculated with the Pearson correlation analysis. **Figure S4.** SNP and indel frequencies distributed differently in individual chromosomes of seven allelic chromosome groups and three genome sets. (A)-(C): calculation based on seven allelic chromosome groups. (D)-(F): calculation based on three genome sets. **Figure S5.** The confirmation of genetic variation. (A): The statistic result of SNP and indel conformation. (B): The confirmation of a SNP CG. (C): The confirmation of a 14 nt deletion. (PDF 775 kb)

## Abbreviations

CDS: Coding DNA sequence
Indel: Insertion and deletion
SNP: Single nucleotide polymorphism
UTR: mRNA Untranslated Region
