# Supplementary material for: The non-random patterns of genetic variation induced by asymmetric somatic hybridization in wheat
Source: BMC Plant Biol. 2018 Oct 17;18:244. doi: 10.1186/s12870-018-1474-3 (PMC6192298; doi:10.1186/s12870-018-1474-3)
Supplement: Supplementary file 1 — Figure S1. The genic distribution of insertions and deletions in unigenes. (A): The frequencies of insertions with sizes form 1 to 10 nt in 5’-UTR, CDS and 3’-UTR. (B): The frequencies of deletions with sizes form 1 to 10 nt in 5’-UTR, CDS and 3’-UTR. Significant difference between CDS and 5′/3’-UTR (labeled *) was measured using the chi-square test of fourfold cross-table analysis. Figure S2. The introgression of exogenous fragment does not induce stronger genetic variation of local chromosome. (A): The transition frequencies of chromosomal arms with and without introgressed exogenous fragments. (B): The transversion frequencies of chromosomal arms with and without introgressed exogenous fragments. (C): The insertion frequencies of chromosomal arms with and without introgressed exogenous fragments. (D): The deletion frequencies of chromosomal arms with and without introgressed exogenous fragments. Total: all unigenes; Mapped: unigenes mapped to different chromosomal arms; Non-introgresed: unigenes mapped to chromosomal arms without exogenous fragments; Introgressed: unigenes mapped to chromosomal arms introgressed with exogenous fragments. P values were obtained via the Student’s t-test. Figure S3. Nucleotide substitutions are not correlative to indels in unigenes mapped to all chromosomes. The correlation was calculated with the Pearson correlation analysis. Figure S4. SNP and indel frequencies distributed differently in individual chromosomes of seven allelic chromosome groups and three genome sets. (A)-(C): calculation based on seven allelic chromosome groups. (D)-(F): calculation based on three genome sets. Figure S5. The confirmation of genetic variation. (A): The statistic result of SNP and indel conformation. (B): The confirmation of a SNP CG. (C): The confirmation of a 14 nt deletion. (PDF 775 kb) [file 12870_2018_1474_MOESM1_ESM.pdf]

Supplemental figure legends

Figure S1. The genic distribution of insertions and deletions in unigenes. (A): The frequencies of insertions with sizes form 1 to 10 nt in 5'-UTR, CDS and 3'-UTR. (B): The frequencies of deletions with sizes form 1 to 10 nt in 5'-UTR, CDS and 3'-UTR. Significant difference between CDS and 5'/3'-UTR (labeled *) was measured using the chi-square test of fourfold cross-table analysis.

Figure S2. The introgression of exogenous fragment does not induce stronger genetic variation of local chromosome. (A): The transition frequencies of chromosomal arms with and without introgressed exogenous fragments. (B): The transversion frequencies of chromosomal arms with and without introgressed exogenous fragments. (C): The insertion frequencies of chromosomal arms with and without introgressed exogenous fragments. (D): The deletion frequencies of chromosomal arms with and without introgressed exogenous fragments. Total: all unigenes; Mapped: unigenes mapped to different chromosomal arms; Non-introgresed: unigenes mapped to chromosomal arms without exogenous fragments; Introgressed: unigenes mapped to chromosomal arms introgressed with exogenous fragments. *P* values were obtained via the Student's *t*-test.

Figure S3. Nucleotide substitutions are not correlative to indels in unigenes mapped to all chromosomes. The correlation was calculated with the Pearson correlation analysis.

Figure S4. SNP and indel frequencies distributed differently in individual chromosomes of seven allelic chromosome groups and three genome sets. (A)-(C): calculation based on seven allelic chromosome

groups. (D)-(F): calculation based on three genome sets.

Figure S5. The confirmation of genetic variation. (A): The statistic result of SNP and indel conformation. (B): The confirmation of a SNP C→G. (C): The confirmation of a 14nt deletion.

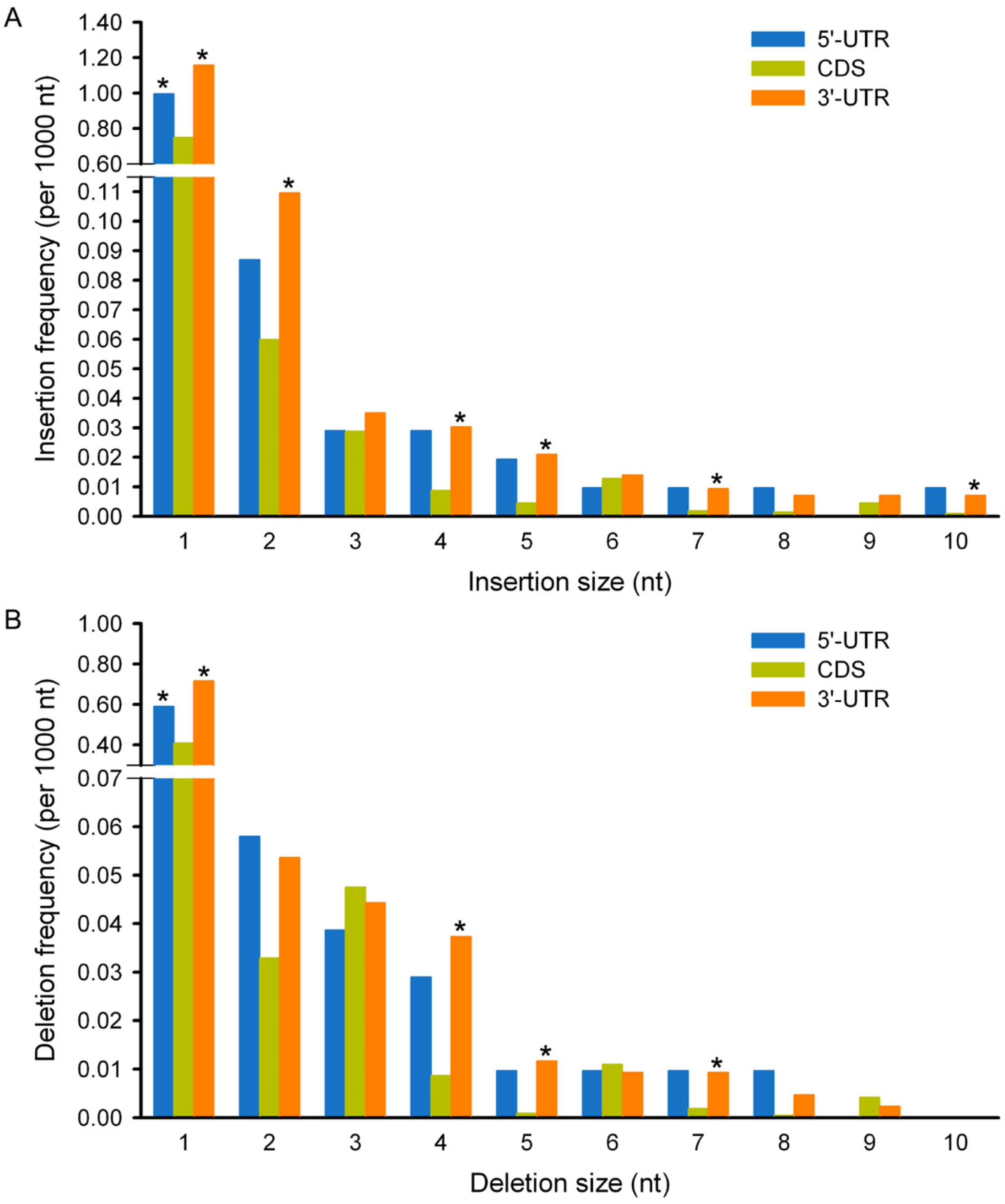
A
B
Insertion frequency (per 1000 nt)
Insertion size (nt)
Deletion frequency (per 1000 nt)
Deletion size (nt)
5'-UTR
CDS
3'-UTR

Figure S1

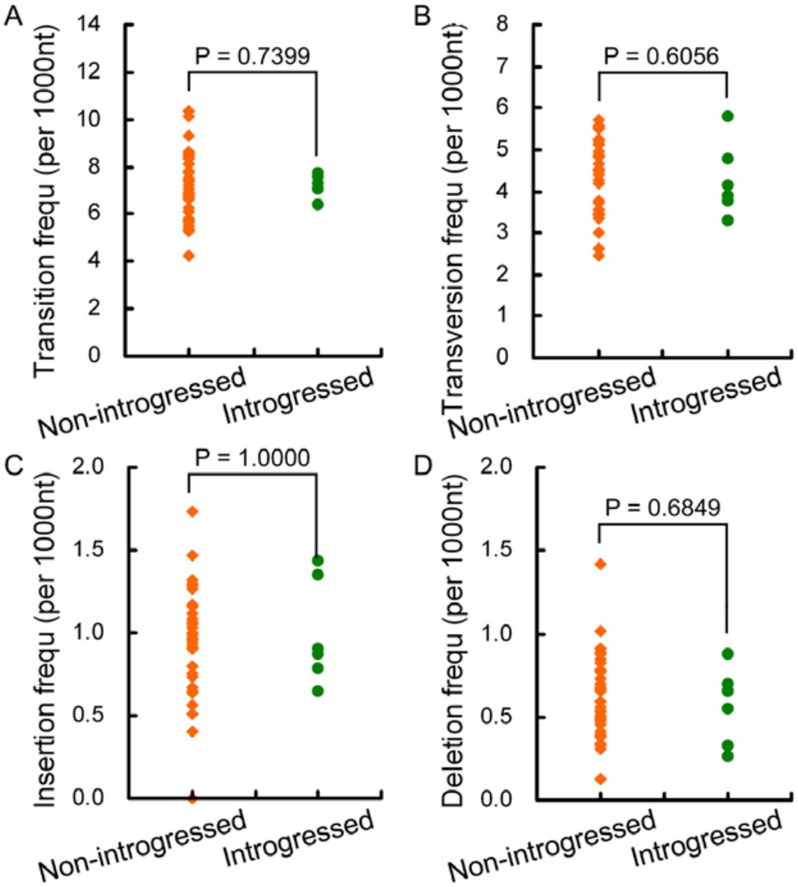
A
Transition frequ (per 1000nt)
P = 0.7399
B
Transversion frequ (per 1000nt)
P = 0.6056
C
Insertion frequ (per 1000nt)
P = 1.0000
D
Deletion frequ (per 1000nt)
P = 0.6849
Non-introgressed
Introgressed

Figure S2

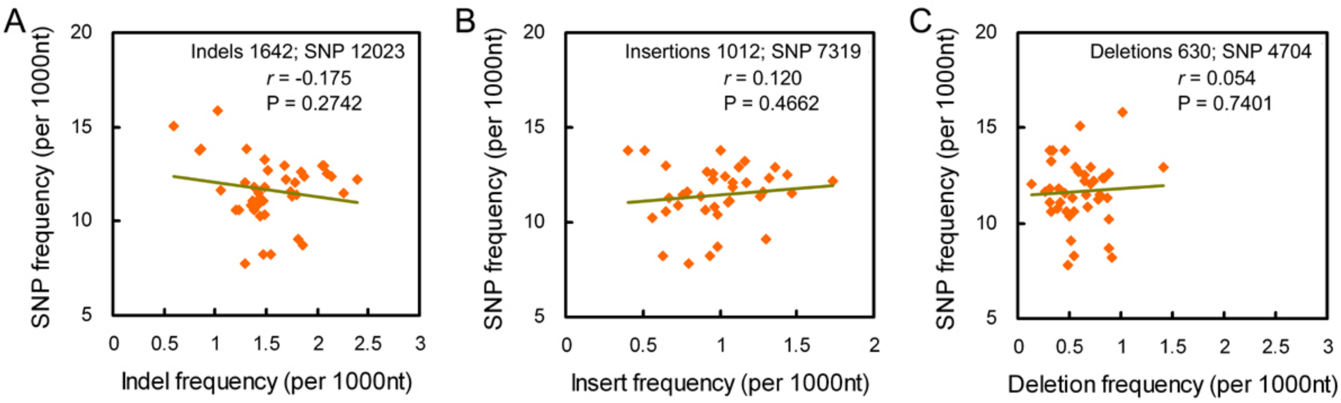
A
B
C
Indels 1642; SNP 12023
r = -0.175
P = 0.2742
Insertions 1012; SNP 7319
r = 0.120
P = 0.4662
Deletions 630; SNP 4704
r = 0.054
P = 0.7401
SNP frequency (per 1000nt)
Indel frequency (per 1000nt)
Insert frequency (per 1000nt)
Deletion frequency (per 1000nt)

Figure S3

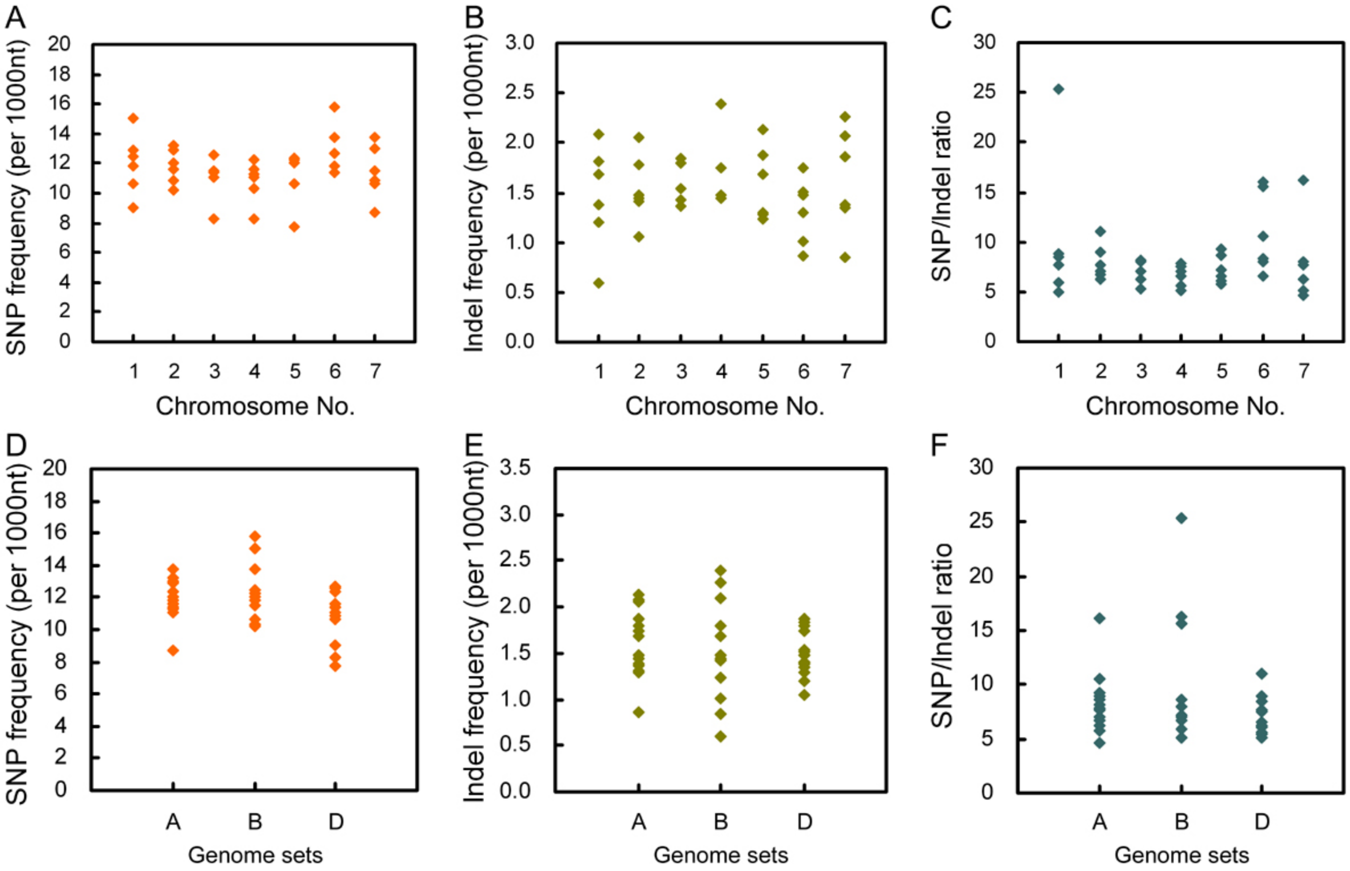

A
SNP frequency (per 1000nt)
0
2
4
6
8
10
12
14
16
18
20
1 2 3 4 5 6 7
Chromosome No.
B
Indel frequency (per 1000nt)
0.0
0.5
1.0
1.5
2.0
2.5
3.0
1 2 3 4 5 6 7
Chromosome No.
C
SNP/Indel ratio
0
5
10
15
20
25
30
1 2 3 4 5 6 7
Chromosome No.
D
SNP frequency (per 1000nt)
0
2
4
6
8
10
12
14
16
18
20
A B D
Genome sets
E
Indel frequency (per 1000nt)
0.0
0.5
1.0
1.5
2.0
2.5
3.0
3.5
A B D
Genome sets
F
SNP/Indel ratio
0
5
10
15
20
25
30
A B D
Genome sets

Figure S4

A

Variation type	In unigenes	Confirmed by PCR	Percentage (%)
SNP	43	42	97.7
Indels	16	16	100.0

B

C

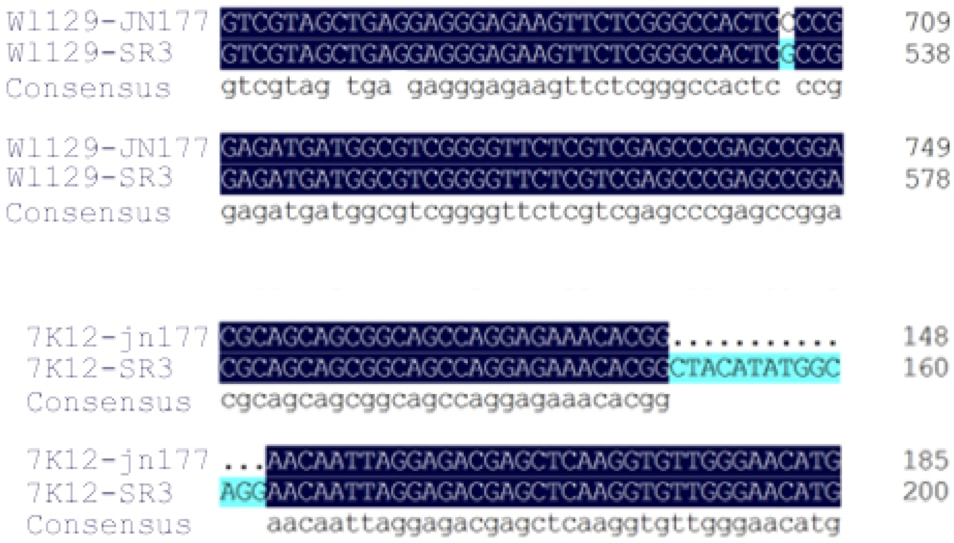

Figure S5
